# Butyrate Inhibits Osteoclast Activity *In Vitro* and Regulates Systemic Inflammation and Bone Healing in a Murine Osteotomy Model Compared to Antibiotic-Treated Mice

**DOI:** 10.1155/2021/8817421

**Published:** 2021-12-10

**Authors:** Alexandra Wallimann, Walker Magrath, Brenna Pugliese, Nino Stocker, Patrick Westermann, Anja Heider, Dominic Gehweiler, Stephan Zeiter, Marcus J. Claesson, R. Geoff Richards, Cezmi A. Akdis, Christopher J. Hernandez, Liam O'Mahony, Keith Thompson, T. Fintan Moriarty

**Affiliations:** ^1^AO Research Institute Davos, Davos, Switzerland; ^2^Swiss Institute of Allergy and Asthma Research (SIAF), University of Zurich, Davos, Switzerland; ^3^SeqBiome Ltd., 3 Castleheights, Bandon, County Cork, Ireland; ^4^Sibley School of Mechanical and Aerospace Engineering, Cornell University, Ithaca, NY, USA; ^5^Hospital for Special Surgery, New York, NY, USA; ^6^Departments of Medicine and Microbiology, APC Microbiome Ireland, University College Cork, College Road, Cork, Ireland

## Abstract

Short-chain fatty acids (SCFAs) produced by the gut microbiota have previously been demonstrated to play a role in numerous chronic inflammatory diseases and to be key mediators in the gut-bone signaling axis. However, the role of SCFAs in bone fracture healing and its impact on systemic inflammation during the regeneration process has not been extensively investigated yet. The aim of this study was to first determine the effects of the SCFA butyrate on key cells involved in fracture healing *in vitro*, namely, osteoclasts and mesenchymal stromal cells (MSCs), and second, to assess if butyrate supplementation or antibiotic therapy impacts bone healing, systemic immune status, and inflammation levels in a murine osteotomy model. Butyrate significantly reduced osteoclast formation and resorption activity in a dose-dependent manner and displayed a trend for increased calcium deposits in MSC cultures. Numerous genes associated with osteoclast differentiation were differentially expressed in osteoclast precursor cells upon butyrate exposure. *In vivo*, antibiotic-treated mice showed reduced SCFA levels in the cecum, as well as a distinct gut microbiome composition. Furthermore, circulating proinflammatory TNF*α*, IL-17a, and IL-17f levels, and bone preserving osteoprotegerin (OPG), were increased in antibiotic-treated mice compared to controls. Antibiotic-treated mice also displayed a trend towards delayed bone healing as revealed by reduced mineral apposition at the defect site and higher circulating levels of the bone turnover marker PINP. Butyrate supplementation resulted in a lower abundance of monocyte/macrophages in the bone marrow, as well as reduced circulating proinflammatory IL-6 levels compared to antibiotic- and control-treated mice. In conclusion, this study supports our hypothesis that SCFAs, in particular butyrate, are important contributors to successful bone healing by modulating key cells involved in fracture healing as well as systemic inflammation and immune responses.

## 1. Introduction

The gut microbiota, comprising bacteria, archaea, viruses, and fungi, has been shown to have a significant role in human health through regulation of host physiology and metabolism [1]. Disruptions in the gut microbiota, as may occur through antibiotic therapy, stress, or an unbalanced diet [2], have been linked with numerous diseases including inflammatory bowel disease [3], metabolic syndrome [4], asthma [5], cardiovascular diseases [6], and bone-associated pathologies, such as osteoporosis [7, 8] and osteoarthritis [9, 10]. Conversely, positive interventions, in the form of prebiotics, probiotics, or postbiotics, have proven health benefits to the host if administered in appropriate amounts [11, 12]. Many of the beneficial effects of the microbiota are mediated by the postbiotic short-chain fatty acids (SCFAs) acetate, propionate, butyrate, and valerate, which are generated by the gut bacteria upon fermentation of dietary fibers [13]. These postbiotics have been extensively studied in relation to chronic inflammatory diseases due to their ability to induce regulatory T cell (Treg) differentiation [14] and secretion of anti-inflammatory cytokines such as IL-10 and TGF*β*, while inhibiting secretion of proinflammatory cytokines, such as IFN*γ*, TNF*α*, IL-1*β*, IL-6, and IL-8 [15]. However, to date, they have been much less studied in relation to bone healing.

Probiotic bacteria themselves have been shown to prevent bone loss by promoting anti-inflammatory immune responses, by increasing mineral absorption in the gut, and through production of endocrine bone signaling factors (e.g., incretins and serotonin) [16]. For example, rats supplemented with the probiotic *Bifidobacterium longum* ATCC 15707 showed increased calcium and magnesium content in their bones [17] and exopolysaccharides from *Bifidobacterium longum 35624®* were shown to inhibit osteoclast formation by a TLR2-dependent mechanism [18]. Another prominent probiotic strain, *Lactobacillus reuteri* ATCC PTA 6475, was shown to prevent ovariectomy-induced bone loss by suppressing CD4+ T cell expansion in bone marrow [19] and to prevent postantibiotic bone loss by reducing microbial dysbiosis and restoring barrier function in the intestine [20]. In fact, broad-spectrum antibiotics followed by four weeks of recovery led to dysbiosis and reduced trabecular femoral bone density, which was dependent on lymphocytes [20, 21].

Although several studies have already investigated the role of the gut microbiota in preventing bone loss, only a limited number of studies have focused on its role in bone fracture healing. Bone healing complications, including delayed healing or nonunions, occur in 5-10% of all long-bone fractures, which lead to pain and functional impairment [22, 23]. Chronic, nonresolving inflammation is a reason for impaired bone healing [24]. Immune cells, including T cells, B cells, and monocytes/macrophages, are crucial players during the fracture healing process and can affect osteoclast formation and activity. The increased prevalence of proinflammatory CD8+/CD57+ T cells in peripheral blood was significantly correlated with delayed bone fracture healing in humans [25]. CD4+ T cells present a heterogenous population with different subpopulations including Th17 and Treg cells. Secretion of proinflammatory IL-17 from Th17 cells is known to stimulate osteoclast formation, whereas increased Treg cell numbers were correlated with higher bone mass and decreased bone resorption in mice [26, 27]. B cells have been shown to secrete OPG, a factor regulating osteoclast differentiation and activity [26]. Circulating CD14+ monocytes/macrophages can serve as osteoclast precursors cells, which migrate to bone to further differentiate to osteoclasts [28, 29].

Two studies recently highlighted the potential of probiotics in bone healing. *Bifidobacterium adolescentis* [30] and *Akkermansia muciniphila* [31] were shown to accelerate bone healing in mice by modulating levels of systemic inflammatory cytokines and gut-epithelial barrier function. Due to the broad effect of SCFAs on different cell types involved in fracture healing, such as mesenchymal stromal cells (MSCs), osteoclasts, and fibroblasts [32], and also their ability to modulate immunity, there is considerable potential of not only probiotics but also postbiotics, in affecting bone healing. However, the role of SCFAs in affecting bone healing and its impact on systemic inflammation during the regeneration process has not been investigated to date.

The aim of this study was to first investigate the effects of SCFA butyrate on key cells involved in fracture healing *in vitro* and, second, to assess if butyrate supplementation impacts bone healing and systemic immune and inflammation levels in a murine osteotomy model. Deficiency in SCFA production was induced by rifampicin and levofloxacin treatment, a common antibiotic regimen administered to fracture patients with staphylococcal bone infections.

## 2. Material and Methods

### 2.1. PBMC Isolation and Osteoclast Differentiation Assay

Peripheral blood mononuclear cells (PBMCs) were isolated from healthy human donors (*n* = 3) via density gradient centrifugation using Histopaque®-1077 reagent (Sigma-Aldrich, Merck KGaA, Darmstadt, Germany) and centrifugation at 800 g for 20 min at room temperature (RT). PBMCs were collected and further cultured under standard conditions (37°C, 5% CO_2_) in *α*MEM supplemented with 10% (*v*/*v*) fetal bovine serum (FBS; Gibco), 100 U/mL penicillin, 100 *μ*g/mL streptomycin, and 20 ng/mL of recombinant human macrophage-colony stimulating factor (M-CSF; R&D Systems, Abingdon, UK) to induce differentiation of osteoclast precursor cells (monocytes/macrophages). Fresh cytokines were added every 2 days, and adherent M-CSF-dependent osteoclast precursors were detached after 5-7 days using trypsin-EDTA solution (Gibco) and a cell scraper, which were then plated into 96-well plates at 1 × 10^4^ cells/well. Recombinant murine receptor activator of NF*κ*B ligand (RANKL; 10 ng/mL; R&D Systems) was added to the cultures to induce the fusion of osteoclast precursors into multinucleated osteoclasts. Additionally, 0.1 mM, 0.25 mM, 0.5 mM, and 1 mM of SCFAs acetate, propionate, butyrate, or valerate were added to investigate the effect of SCFAs on osteoclast differentiation. Likewise, 0.5 *μ*g/mL, 5 *μ*g/mL, and 50 *μ*g/mL rifampicin and/or levofloxacin were added to investigate the effect of antibiotics on osteoclast formation. Medium, cytokines, SCFAs, and antibiotics were replaced every 2 days, and after 7-10 days, cells were fixed using a 4% formaldehyde solution in PBS for 15 min at RT. To determine osteoclast formation, cells were stained using a tartrate-resistant acid phosphatase (TRAcP) staining kit (Sigma-Aldrich, Buchs, Switzerland). Multinucleated (≥3 nuclei) TRAcP-positive cells were classified as osteoclasts, and the total number of osteoclasts per well was quantified using a Zeiss Axiovert A1 light microscope (Zeiss).

### 2.2. Resorption Assay

To assess the effects of butyrate on mature osteoclasts, osteoclasts were first generated by seeding 300,000 osteoclast precursor cells/well in 6-well plates and stimulated with M-CSF and RANKL as described above. Once cell fusion was observed (typically 3-4 days following RANKL addition), mature osteoclasts were detached by trypsinization and gentle scraping, then resuspended into a complete medium (containing 20 ng/mL M-CSF and 10 ng/mL RANKL; 2 mL/well) before seeding into Osteoassay 96-well plates (Corning) at 100 *μ*L cell suspension/well. After allowing the cells to attach to the Osteoassay substrate for 4 hours, 0.1 mM, 0.25 mM, 0.5 mM, or 1 mM butyrate was added to the respective wells. After 72 hours, cells were washed twice with PBS and were then fixed using 4% (*v*/*v*) formaldehyde solution for 15 min at RT. To assess osteoclast resorption, the cells were removed using 10% (*v*/*v*) bleach, and a von Kossa silver nitrate staining was performed.

### 2.3. Cell Viability Assay

Effects of butyrate on cell viability of osteoclast precursors and MSCs were assessed using CellTiter-Blue reagent (Promega AG, Dübendorf, Switzerland), according to the manufacturer's instructions.

### 2.4. Functional Osteoclast Formation Assays

To check whether GPR43 and GPR183 are required for osteoclast formation, human osteoclast precursor cells were isolated and cultured as described above but additionally treated with either 75 nM, 750 nM, or 7500 nM of GPR43 (FFA2) agonist (Sigma-Aldrich) or 5 nM, 50 nM, or 500 nM of inverse GPR183 agonist (GSK682753A, MedChemExpress). Compounds were dissolved in dimethylsulfoxide (DMSO, Sigma). Osteoclast quantification was performed with the TRAcP staining kit, as described above.

### 2.5. Isolation and Expansion of Human Bone Marrow-Derived Mesenchymal Stromal Cells

Human bone marrow aspirates were obtained with informed consent of all donors and with full approval from the Ethics Committee of the University of Freiburg Medical Centre (EK-Freiburg: 135/14) and the ethical commission of Graubünden (KEK-ZH-NR: 2016-00141). Human bone marrow-derived mesenchymal stromal cells (BM-MSCs) were isolated by density gradient centrifugation using Histopaque-1077 and cultured as previously described [33, 34]. BM-MSCs were seeded at 3 × 10^3^ cells/cm^2^ in *α*-MEM supplemented with 10% (*v*/*v*) MSC-qualified FBS (Sera-Plus, PAN-Biotec GmbH, Aidenbach, Germany), 100 U/mL penicillin, 100 *μ*g/mL streptomycin, and 5 ng/mL basic fibroblast growth factor-2 (bFGF-2; Fitzgerald Industries, USA), under standard conditions of 37°C and 5% CO_2_ in a humidified atmosphere. For osteogenesis and chondrogenesis assays, culture-expanded MSCs were used up to passage 5.

### 2.6. In Vitro MSC Osteogenic Differentiation Assay

To assess the impacts of butyrate on MSC osteogenic differentiation, human BM-MSCs (*n* = 3) were plated at 3 × 10^4^ cells/well on Thermanox coverslips in 24-well plates and grown until confluent. At this point, cell monolayers were treated with either control medium (1 g/L glucose DMEM, 10% FBS, 100 U/mL penicillin, and 100 *μ*g/mL streptomycin) or osteogenic medium: control medium additionally supplemented with 50 *μ*g/mL ascorbic acid 2-phosphate (AA2P; Sigma), 5 mM *β*-glycerophosphate (Sigma), and 10 nM dexamethasone (Sigma). Culture medium was exchanged three times per week, and the cells were cultured for a total period of 28 days. Butyrate (0.5 mM) was added to the wells and was replenished at each medium change. Cells were then washed twice with PBS, fixed with 4% formaldehyde for 15 min at RT, and washed three times in distilled water. The cell monolayer was then stained using a 40 mM solution of Alizarin red solution (pH 4.2) for 1 hour on a rocking platform. The cells were then washed five times with distilled water, and Alizarin red staining was imaged using an inverted light microscope.

To quantify mineralization, Alizarin red was extracted by incubating in 10% (*v*/*v*) acetic acid at RT for 30 min. After removal of the monolayer by scraping, the samples were then heated at 85°C for 10 min and cooled on ice. After centrifugation at 13,000 g for 10 min the supernatant was collected, then the pH was altered to 4.3 using 10% (*v*/*v*) ammonium hydroxide. Quantification was assessed in comparison to known Alizarin red concentrations by measuring the absorbance of the standards/samples at 405 nm with a Multiskan™ GO 3.2 microplate spectrophotometer and analyzed using SkanIt™ Software (Thermo Fisher Scientific, Waltham, MA, USA).

### 2.7. In Vitro MSC Chondrogenic Differentiation Assay

Human BM-MSCs (*n* = 5) were culture expanded as described above. BM-MSCs were then harvested using trypsin-EDTA (Gibco) and resuspended in chondropermissive media consisting of Dulbecco's Modified Eagle Serum (DMEM) 4.5 g/L glucose, 50 *μ*g/mL AA2P, 1% (*v*/*v*) nonessential amino acids, 100 U/mL penicillin, 100 *μ*g/mL streptomycin, 1% (*v*/*v*) ITS supplement (Corning), and 100 nM dexamethasone. Cells were seeded in quadruplicates into a 96-well V-bottomed plate at a density of 2 × 10^5^ cells/well. The plate was then centrifuged (400 g, 5 min), and pellets were allowed to form for 24 hours. Fresh chondropermissive medium or chondrogenic medium (chondropermissive medium supplemented with 10 ng/mL TGF*β*1 (Fitzgerald Industries)) was then added to the cell pellets (day 0). To assess the impacts of SCFA supplementation on chondrogenesis of MSCs, pellets cultured under chondropermissive and chondrogenic conditions were also treated with 0.5 mM butyrate. Growth medium was replaced every 2-3 days, with conditioned medium collected and stored for subsequent assessment of sulphated glycosaminoglycan (sGAG) content. Cell pellets were harvested at day 24. Two pellets per condition were subsequently processed for histological assessment, and the remaining 2 pellets were processed for sGAG content analysis. Chondrogenic differentiation was assessed using Safranin O/Fast Green to visualize proteoglycans and collagen. Pellets were fixed using 4% formaldehyde then dehydrated in an ascending ethanol series prior to embedding in paraffin. Sections cut at 6 *μ*m were then stained with Safranin O/Fast Green and visualized using light microscopy. Content of sGAG content in the pellets was assessed using 1,9-dimethyl-methylene blue (DMMB), following overnight digestion of the pellets at 56°C in 0.5 mg/mL Proteinase K (Roche) solution. Absorbance was measured immediately at 535 nm using a Victor^3^ Microtitre plate reader (Perkin Elmer®), using a standard curve of known concentrations of chondroitin sulphate. Results were expressed after normalization to DNA content of cell pellets using Hoechst 33258 and calf thymus DNA as a standard.

### 2.8. Bulk RNA Sequencing

To characterize gene expression changes in osteoclast precursor cells upon butyrate stimulation, PBMCs from five healthy human donors were isolated as described above. Magnetic-activated cell sorting (MACS) using CD14 MicroBeads (130-050-201, Miltenyi Biotec), positive selection columns (MS+ column 120-000-472, Miltenyi Biotec), and OctoMACS™ separator (Miltenyi Biotec) was performed on isolated PBMCs to recover CD14+ monocytes/macrophages. Purity of positively selected CD14+ monocytes/macrophages was assessed using flow cytometry and was ≥95%. CD14+ monocytes/macrophages were further cultured in *α*MEM supplemented with 10% (*v*/*v*) FBS, 100 U/mL penicillin, 100 *μ*g/mL streptomycin, and 20 ng/mL of human M-CSF until confluency. After 5-7 days, adherent M-CSF-dependent osteoclast precursors were detached using trypsin-EDTA solution and a cell scraper and subsequently seeded in 6-well plates (3 × 10^5^ cells/2 mL) and stimulated with 20 ng/mL human M-CSF. The following day, 20 ng/mL murine RANKL and 0.5 mM butyrate were added to the osteoclast precursor cells. After 6 h and 24 h of stimulation with 0.5 mM butyrate and RANKL, whole RNA was isolated by means of RNeasy® plus micro kit (Qiagen, Hilden, Germany) according to manufacturer's instructions. Purity and integrity of isolated RNA were assessed using a spectrophotometer (NanoDrop, Thermo Fisher Scientific) and Agilent 2200 TapeStation (Agilent Technologies, Waldbronn, Germany), respectively. RNA library processing (poly A selection) and Illumina Hiseq single end (150 bp) sequencing were performed at the Functional Genomics Center Zurich. Data was analyzed within the sushi data analysis framework launched by Functional Genomics Center Zurich and by MetaCore software (Clarivate™ Analytics). False discovery rate (FDR) threshold was set to <0.01 and fold change ratio to ±0.5.

### 2.9. In Vivo Experimental Design

The *in vivo* experiment was approved by the Tierversuchskommission Graubünden (Approval Nr. 2019_25). Healthy male C57BL/6J mice (*n* = 55, including reserves), free of orthopedic disease, were obtained from Charles River (Germany). Mice were acclimatized for two weeks and were housed at a 12 h dark/12 h light cycle in groups of 2-6 in individually ventilated cages. Single housing was deemed necessary for certain mice due to aggression and hierarchy fights within cages. Mice were fed ad libitum and had constant access to water. Mice were randomly allocated to one of the following experimental groups (*n* = 12 per group): group 1, the control group, was orally gavaged with vehicle (sterile water) in the morning and afternoon (8 hours later); group 2 was orally gavaged with the vehicle in the morning and with 30 mM butyrate in the afternoon; group 3 received a 25 mg/kg rifampicin plus 20 mg/kg levofloxacin (Rif + Levo) antibiotic mixture in the morning and vehicle in the afternoon. An additional fourth group was administered Rif + Levo antibiotic mixture in the morning and 30 mM butyrate in the afternoon. However, after suffering from a greater than anticipated drop-out rate, the results of this experiment are not presented (see Discussion). Mice were orally gavaged with the corresponding treatment (administration volume 200 *μ*L) for five consecutive days per week during the whole study duration (21 days), starting the gavage the morning of osteotomy. At 20 weeks of age, mice were placed under general anesthesia and a 0.44 mm femoral osteotomy was performed, under aseptic conditions, in the left hind limb using a 4-hole jig and Gigli wire (Mouse Fix Drill & Saw guide, RIS.301.107). A 4-hole polyether ether ketone (PEEK) plate (RISystem MouseFix Plate 4-hole, PEEK RIS.601.001), 0.31 mm drill bit (RIS.592.202), and 4 self-cutting angular stable screws (MouseFix Screw, length 2 mm, RIS. 401.100) were used to fix the osteotomy. Mice were operated group by group, and surgeons were therefore not blinded. Mice were subcutaneously injected with calcein green (5 mg/kg) at 10 days and with xylenol orange (90 mg/kg) at 18 days after osteotomy to investigate calcium apposition retrospectively.

### 2.10. Anesthesia, Analgesia, and Euthanasia

Mice were anesthetised with sevoflurane (1.5-3% in O_2_, flow rate 0.6-0.8 L/min) during the surgery and CT scans, and before euthanasia, which was done by cervical dislocation. Intraoperative analgesia consisted of buprenorphine (1 : 10 dilution of 0.3 mg/mL solution; 0.1 mL subcutaneously (*s.c.*)) and carprofen (1 : 10 dilution of 50 mg/mL solution; 0.1 mL *s.c.*) immediately after anesthetic induction. To prevent loss of body temperature, the mice were placed on a temperature-controlled heating mat pre- and intraoperatively. Postoperative analgesia consisted of tramadol added to the drinking water (25 mg/L, 1 drop per 100 mL tap water) for 7 days.

### 2.11. MicroCT Imaging

MicroCT scans of the operated femora were performed using VivaCT40 (SCANCO Medical AG, Brüttisellen, Switzerland). Images were acquired using a voxel resolution of 10.5 *μ*m and a beam energy and intensity of 70 kVp and 114 *μ*A, respectively. Scans were performed at three different timepoints: immediately following surgery, and at 10 and 21 days postsurgery (at euthanasia).

### 2.12. Blood Collection and Serum Preparation

Blood was collected on the day of osteotomy (preoperatively) and at 10 days postoperatively from the lateral tail vein and at 21 days (at euthanasia) from the retrobulbar vessels. The collected blood was allowed to clot at RT for 30 min, then centrifuged for 10 min at 6000 g at RT. Serum was collected and frozen at -20°C until further analysis.

### 2.13. Quantification of Serum Inflammatory and Bone Turnover Markers

Rat/Mouse PINP EIA kit (ids®, UK) was used to assess levels of type I procollagen (PINP) in mouse serum, according to the manufacturer's protocol. Data were collected using a Multiskan™ GO 3.2 microplate spectrophotometer and analyzed using SkanIt™ Software. A V-PLEX Mouse Proinflammatory Panel kit (Meso Scale Diagnostics, Rockville, MD, USA) was used to test a panel of inflammatory markers including IL-6, IL-10, IL-1*β*, TNF*α*, and KC/GRO, in the serum of mice. The assay was performed according to the manufacturer's protocol.

### 2.14. Olink® Targeted Proteomics

An Olink® Target 96 Mouse Exploratory panel (Olink Proteomics, SE-751 83 Uppsala, Sweden) was used to assess 92 protein biomarkers in the murine serum samples. Data were analyzed using the Olink® Insights Stat Analysis app and with GraphPad Prism software (GraphPad Software 8.1.0, Inc., La Jolla, CA, USA).

### 2.15. Histological Processing and Morphometric Analysis

Operated femora (*n* = 5 per group), with the 4-hole PEEK-plate attached, were fixed in methanol and subsequently embedded in methyl methacrylate (MMA). Slices of 50-70 *μ*m were cut and imaged on an Olympus BX63F light microscope. Upon fluorescence imaging, slices were stained with a 15% (*v*/*v*) Giemsa and a 1% (*v*/*v*) Eosin solution and imaged with an Olympus BX63F light microscope. Images of fluorescent mouse femora were analyzed using ImageJ (NIH, Bethesda, USA) and scaled using a 500 *μ*m scale bar. The region of interest (ROI) was a rectangular area surrounding the tissue between the two middle screws and the osteotomy. The red and green channels were used to quantify the mean fluorescent intensity (mean pixel value) of xylenol orange and calcein green, respectively, in each image.

### 2.16. Biomechanical Testing of Femora

To measure stiffness of the newly formed callus and the mechanical properties of unoperated femora, four-point bending (destructive testing) was performed. PEEK implants were carefully dissected from operated femora (*n* = 7 per group), and femora were moistened with Ringer's solution, wrapped in gauze, and frozen at -20°C until testing was performed. Unoperated, contralateral femora were collected and stored in the same manner. Duration of frozen storage was kept consistent for all samples. On the day of measurement, mouse femora were removed from Ringer's solutions and nonbone tissue was carefully removed from each sample. An Instron® 5866 machine (Norwood MA, US) with a 100 N loadcell was used for the mechanical testing. All samples were placed in the same orientation on two bending points. Preload between 0 and 0.05 N was applied to the samples, and testing was performed with a speed of 0.5 mm/min. Measurement was aborted as soon as femora samples broke. Plots of force versus displacement were derived from the four-point bending test. Failure load was extrapolated from each curve.

### 2.17. 16S rRNA Sequencing and Data Analysis

Cecal content of mice was harvested on the day of euthanasia and frozen at -20°C until further processing. DNA of cecal content of mice was isolated using QIAmp® PowerFecal Pro DNA kit (Qiagen). Samples were sequenced and analyzed by SeqBiome Ltd. (County Cork, Ireland). DADA2 R package was used for data analysis using the SILVA 138 as reference database for taxonomy assignment.

### 2.18. SCFA Measurement in Murine Cecal Water

Cecal water from residual cecal content, which was not used for DNA isolation, was prepared to measure SCFAs. Sulfuric acid (0.15 mM) was added to cecal content in a ratio of 1 mL per 0.3 g. Samples were rigorously vortexed and then centrifuged at 14,000 g for 30 min at 4°C. Supernatant was collected and again centrifuged at the same speed. Samples were sequentially filtered through a 0.45 *μ*m and 0.2 *μ*m filter. The filtered supernatants were analyzed on an ACQUITY UPLC H-Class Bio System (Waters Corp, Milford, MA, USA). The separation was carried out on an Aminex HPX-87H ion exchange column (300 mm × 7.8 mm, 9 *μ*m particle, Bio-Rad Laboratories Inc.) together with a Micro-Guard Cation H+ refill cartridge (Bio-Rad Laboratories Inc.) at a flow rate of 0.35 mL/min at 40°C with 10 mmol/L H_2_SO_4_ as an eluent solution. Injection volume was 20 *μ*L, and the detection wavelength was 210 nm. The samples were quantified in relation to standards measured in parallel.

### 2.19. Flow Cytometric Analysis

Spleen, inguinal lymph node (iLN) from the operated site, and tibial bone marrow were collected on the day of euthanasia. Single-cell suspensions were prepared from all tissues using a 40 *μ*m cell strainer, followed by staining for the following surface markers: PE anti-mouse CD3 antibody (clone: 17A2, isotype: rat IgG2b), Alexa Fluor® 700 anti-mouse CD4 antibody (clone: GK1.5, isotype: rat IgG2b, *κ*), PE/Dazzle™ 594 anti-mouse CD8a antibody (clone:53-6.7, isotype: Rat IgG2a, *κ*), Brilliant Violet 510™ anti-mouse CD45 (clone:30-F11, isotype: rat IgG2b, *κ*), PE-Cy5 anti-mouse CD19 (clone: 6D5, isotype: rat IgGa, *κ*), and FITC anti-mouse CD14 (clone: Sa14-2, isotype: rat IgG2a, *κ*). Cell viability was assessed using fixable viability dye eFluor™ 780. Antibodies were all purchased from BioLegend and the viability dye from eBioscience. Samples were acquired using a BD FACSAria™ III Cell Sorter (BD Biosciences, New Jersey, US) and analyzed with Kaluza Software (Beckman Coulter GmbH, Germany).

### 2.20. Statistical Analysis

Data is reported as mean ± SEM unless stated otherwise. One-way ANOVA was used to determine statistical significance between experimental groups, using Tukey's post hoc analysis. Threshold for statistical significance was *p* < 0.05. Unless stated otherwise, all analyses were performed using GraphPad Prism software (GraphPad Software 8.1.0, Inc., La Jolla, CA, USA).

## 3. Results

### 3.1. Butyrate Inhibits Formation and Resorption Activity of Human Osteoclasts and Affects Osteogenic Differentiation of Human MSCs

**Figure 1:**
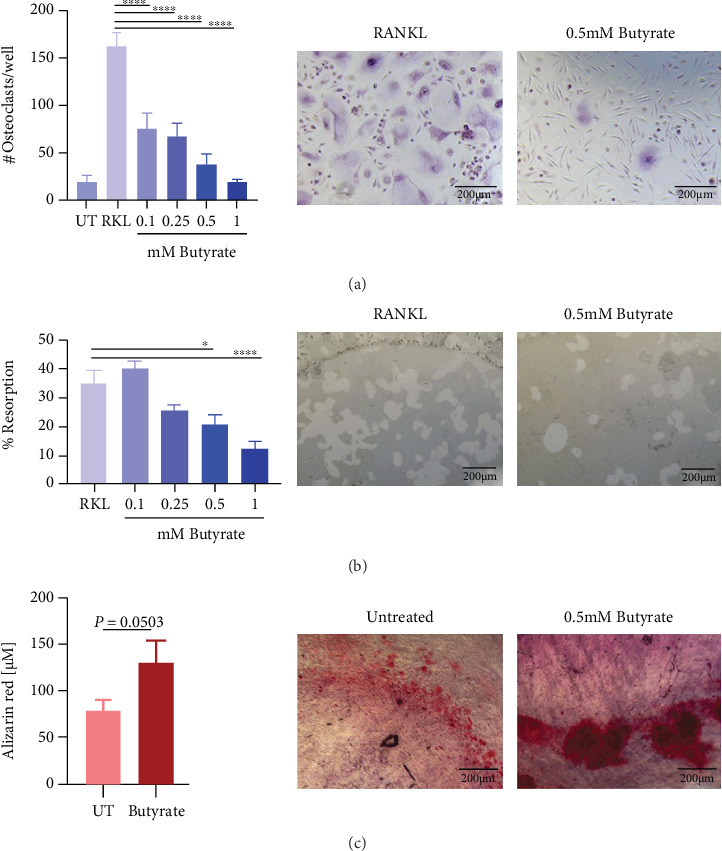
Butyrate inhibits human osteoclast formation and resorption. Impact of butyrate on cells involved in bone healing. (a) Osteoclast precursor cells were cultured with 20 ng/mL M-CSF and 10 ng/mL RANKL and with 0.1 mM, 0.25 mM, 0.5 mM, or 1 mM butyrate, respectively. Osteoclast formation was quantified by means of TRAcP staining. Data shown are means (*n* = 3 independent donors, triplicates per donor were used) ± SEM. Representative images showing TRAcP-stained osteoclasts in the absence (left image) and presence of 0.5 mM butyrate (right image). (b) Mature osteoclasts were cultured on a hydroxyapatite-coated plate in the presence and absence of butyrate with concentrations as indicated above. Resorption areas were quantified by means of von Kossa staining. Data shown are means ± SEM (*n* = 1 donor, performed in triplicate, and two pictures per well were taken and quantified). Representative images showing resorbed areas of hydroxyapatite-coated wells in the absence (left image) and presence of 0.5 mM butyrate (right image). (c) MSCs were cultured in osteogenic media and calcium deposits were quantified by means of Alizarin red staining in the absence and presence of 0.5 mM butyrate. Data shown are means (*n* = 3 independent donors) ± SEM. Representative images of Alizarin red staining in the absence (left image) and presence of 0.5 mM butyrate (right image). Scale bar in all images = 200 *μ*m; UT = untreated; RKL = RANKL; ⁣^∗∗∗∗^*p* < 0.0001; ⁣^∗^*p* < 0.05.

To investigate the impact of butyrate on osteoclast formation, osteoclast precursor cells were generated from human PBMCs. These osteoclast precursor cells were treated with RANKL alone or with butyrate at concentrations from 0.1 mM to 1 mM. Quantification of osteoclasts by means of TRAcP staining revealed a significant (*p* < 0.0001) reduction of osteoclast formation in the presence of 0.1 mM (54% reduction), 0.25 mM (59% reduction), 0.5 mM (76% reduction), and 1 mM of butyrate (89% reduction) (Figure 1(a)). Similar inhibitory effects could also be detected with other SCFAs, including acetate, propionate, and valerate (Supplementary Figure 1A). However, the presence of 0.5 *μ*g/mL, 5 *μ*g/mL, and 50 *μ*g/mL rifampicin or levofloxacin did not significantly affect osteoclast formation (Supplementary Figure 1B). The resorption activity of mature osteoclasts was also significantly (*p* < 0.05) reduced in the presence of 0.5 mM (40% reduction) and 1 mM butyrate (66% reduction) (Figure 1(b)). Concentrations below 0.25 mM had no effect on cell viability, and less than 30% reduction was observed at higher concentrations after 72 hours of treatment (Supplementary Figure 1C) without obvious signs of toxicity as seen in Figure 1(a). In contrast to the inhibitory effects of butyrate on osteoclast formation and resorption activity, 0.5 mM butyrate displayed a trend for increased calcium deposits in MSCs cultured in osteogenic media (Figure 1(c), *p* = 0.0503) as revealed by Alizarin red staining. However, no significant change was observed in sGAG content of pellet culture of MSCs in chondrogenic media in the presence of 0.5 mM butyrate (Supplementary Figure 1B). Cell viability of MSCs treated with 0.5 mM butyrate was not significantly affected (Supplementary Figure 1D). Overall, these *in vitro* experiments show that butyrate influences both the differentiation and the activity of bone-resorbing osteoclasts and the osteogenic differentiation of MSCs, in a manner expected to be beneficial for bone remodeling and healing.

### 3.2. Butyrate Affects Pathways and Expression of Genes Relevant for Bone Healing and Osteoclast Differentiation

**Figure 2:**
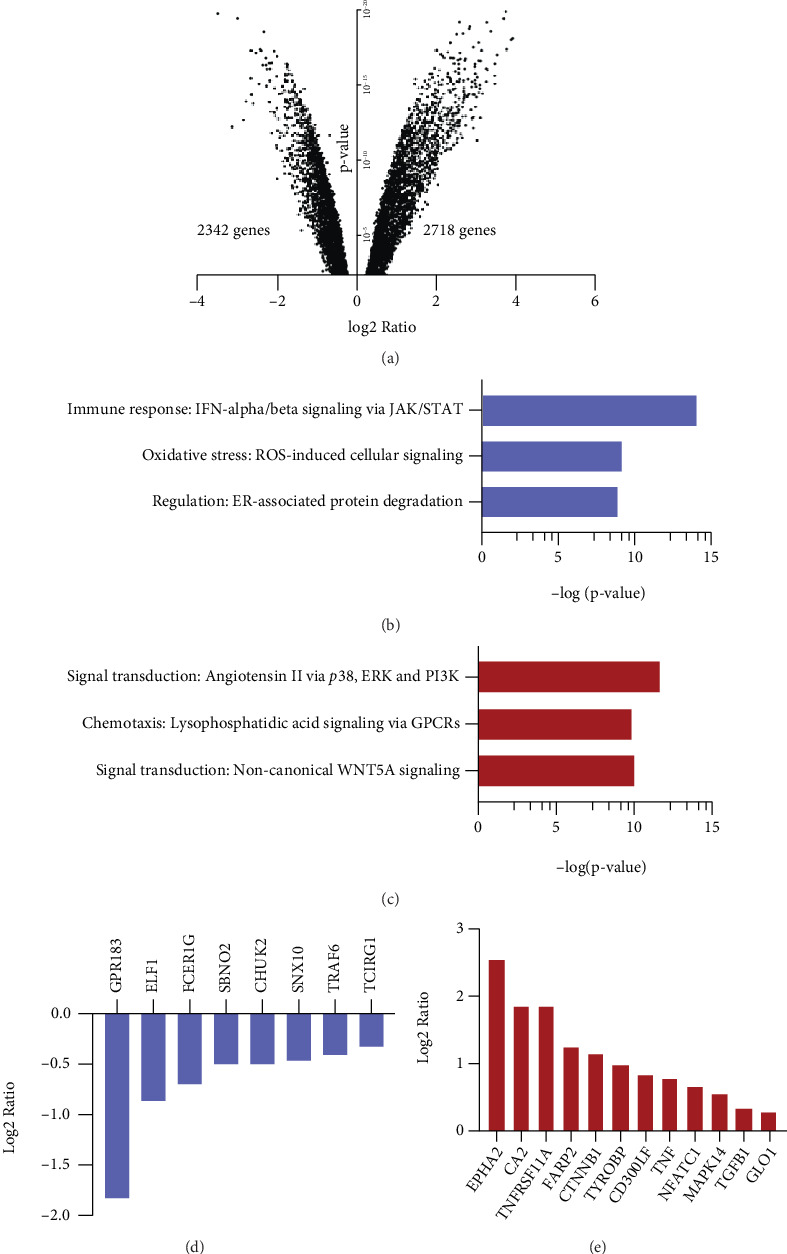
Butyrate regulates pathways and expression of genes relevant for bone healing and osteoclast differentiation. RNA sequencing of human osteoclast precursor cells following 6 h of butyrate treatment. (a) Volcano plot showing number of upregulated and downregulated genes in osteoclast precursor cells following 6 h stimulation with 0.5 mM butyrate. Functional enrichment analysis showing the three most significantly downregulated (b) and upregulated (c) pathways following 6 h of 0.5 mM butyrate treatment. (d) Downregulation and (e) upregulation of genes involved in osteoclast differentiation following 6 h 0.5 mM butyrate treatment. Fold change threshold = 0.5 and FDR threshold < 0.01 for all charts.

By means of bulk RNA sequencing, we further explored potential pathways and genes involved in butyrate-mediated inhibition of osteoclast formation. The transcriptome of osteoclast precursor cells (CD14+ monocytes/macrophages) of five healthy human donors was analyzed in the absence and presence of 0.5 mM butyrate at 6 and 24 hours. Following 6 h of butyrate stimulation, 2718 genes were upregulated and 2342 genes downregulated (Figure 2(a)). Following 24 h of butyrate treatment, only four genes passed the FDR threshold (LAD1, CCR7, HTR2B, and ANKRD1, data not shown). Pathway enrichment analysis by means of MetaCore™ software revealed several pathways relevant for bone healing were significantly altered. Immune response pathway related to IFN-alpha/beta signaling, ROS-induced oxidative stress cellular signaling, and endoplasmic reticulum- (ER-) associated protein degradation were the top three statistically significant downregulated pathways in osteoclast precursor cells upon butyrate stimulation (Figure 2(b)). Upregulated pathways included signal transduction of angiotensin II via p38, ERK, and PI3K, pathways associated with chemotaxis signaling via GPCR, and signal transduction of bone-related WNT5A signaling (Figure 2(c)). As part of GPCR signaling, stimulation of GPR43 with an agonist lead to a significant (*p* < 0.01) reduction in osteoclast formation (Supplementary Figure 2A). DMSO, which served as solvent for the GPR43 agonist, did not influence osteoclast formation (data not shown).

Investigation of individual genes involved in osteoclast differentiation and fusion revealed a significant downregulation of GPR183, ELF1, FCER1G, SBNO2, CHUK, SNX10, TRAF6, and TCIRG1 in butyrate-treated osteoclast precursor cells (Figure 2(d)). Inhibition of GPR183 using a selective inverse agonist (GSK682753A) only slightly affected osteoclast formation (Supplementary Figure 2B). In contrast, EPHA2, CA2, TNFRSF11A (also known as the RANK receptor), FARP2, CTNNB1, TYROBP, CD300LF, TNF, NFATC1, MAPK14, TGFB1, and GLO1 were significantly upregulated in butyrate-treated osteoclast precursor cells (Figure 2(e)). To summarize, butyrate induces marked changes in the transcriptome of osteoclast precursors involving pathways relevant for bone healing and genes crucial for osteoclast differentiation and fusion.

### 3.3. Rifampicin- and Levofloxacin-Treated Mice Show Reduced Cecal SCFA Levels and a Change in Gut Microbiome Composition Compared to Butyrate- and Control-Treated Mice

**Figure 3:**
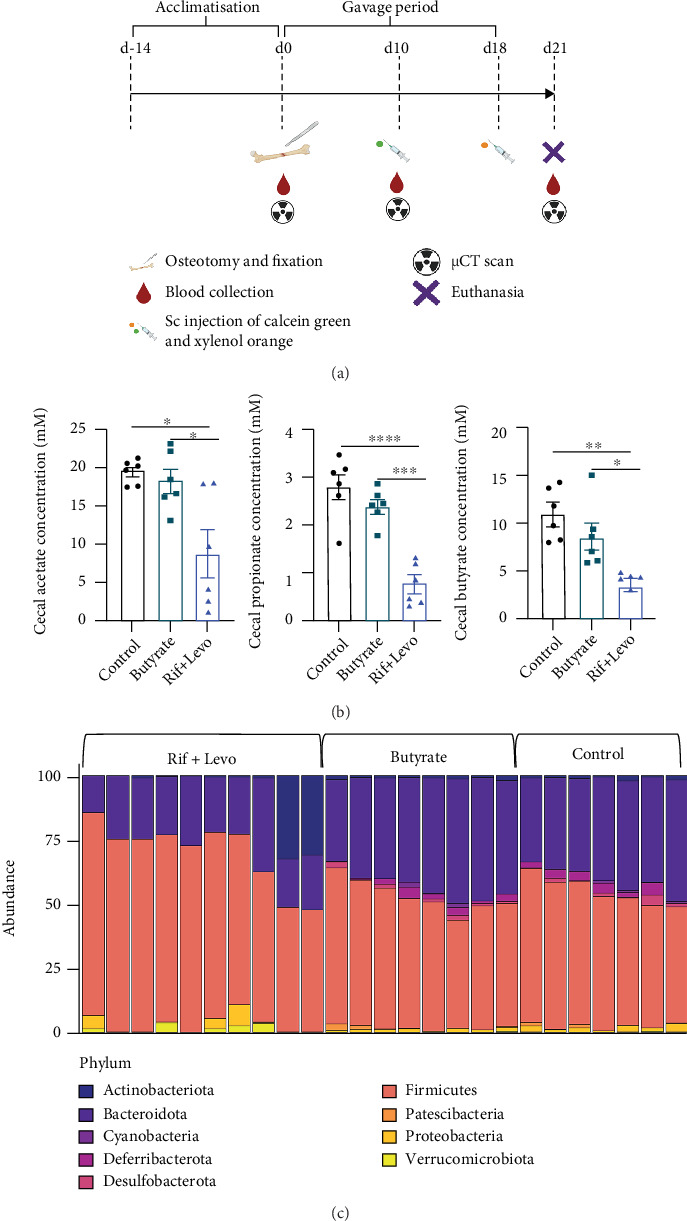
Rifampicin- and levofloxacin-treated mice show reduced cecal SCFA levels and a change in gut microbiome composition compared to butyrate- and control-treated mice. The effect of butyrate on the gut microbiome, systemic immunity, and bone healing was assessed in a murine osteotomy model. (a) Experimental outline of *in vivo* study indicating interventions and time frame. The figure was created with http://BioRender.com/. (b) Cecal concentrations of acetate, propionate, and butyrate were measured by means of UPLC. Data shown are means (*n* = 6) and ±SEM. ⁣^∗∗∗∗^*p* < 0.0001, ⁣^∗∗∗^*p* < 0.001, ⁣^∗∗^*p* < 0.01, and ⁣^∗^*p* < 0.05. (c) DNA of murine cecal content was isolated, and 16s rRNA sequencing was performed to determine microbiome composition. Percent abundance of bacterial phyla in the murine cecum (*n* = 10Rif + Levo-treated animals, *n* = 8 butyrate-treated animals, and *n* = 7 control-treated animals).

Based on our *in vitro* data, which demonstrates the potential beneficial effects of butyrate on key cells involved in bone remodeling and healing, namely, osteoclasts and MSCs, we further investigated this in a murine osteotomy model (overview provided in Figure 3(a)). Control mice were compared with mice receiving oral butyrate supplementation and mice receiving oral Rif + Levo. Rif + Levo-treated mice show up to a three-fold reduction of acetate, propionate, and butyrate in cecal water compared to butyrate- and control-treated mice (Figure 3(b), *p* < 0.05). Butyrate supplementation did not impact the gut microbiome composition of mice compared to controls; however, Rif + Levo administration had marked effects as revealed by Principle Coordinate Analysis (PCoA) using Bray-Curtis distances (Supplementary Figure 2A). Considering bacterial phylum abundance, Rif + Levo-treated mice show higher *Firmicutes* abundance, whereas butyrate- and control-treated mice show higher *Bacteroidata* abundance (Figure 3(c)). Furthermore, Rif + Levo-treated mice show higher relative abundances of *Prevotellaceae*, *Rikenellaceae*, and *Deferribacteraceae* families (Supplementary Figure 2B) and have higher relative proportions of *Clostridiodes* genera compared to control- and butyrate-treated mice (Supplementary Figure 2C). Taken together, butyrate supplementation did not impact gut microbiota or SCFA production; however, administration of an antibiotic regimen involving Rif + Levo strongly reduced SCFA levels in the cecal water and induced a marked change in the gut microbiome composition.

### 3.4. Butyrate Induces a Reduction of Proinflammatory Mediators, Whereas Rifampicin and Levofloxacin Increase Proinflammatory and Bone-Preserving Markers in Serum

**Figure 4:**
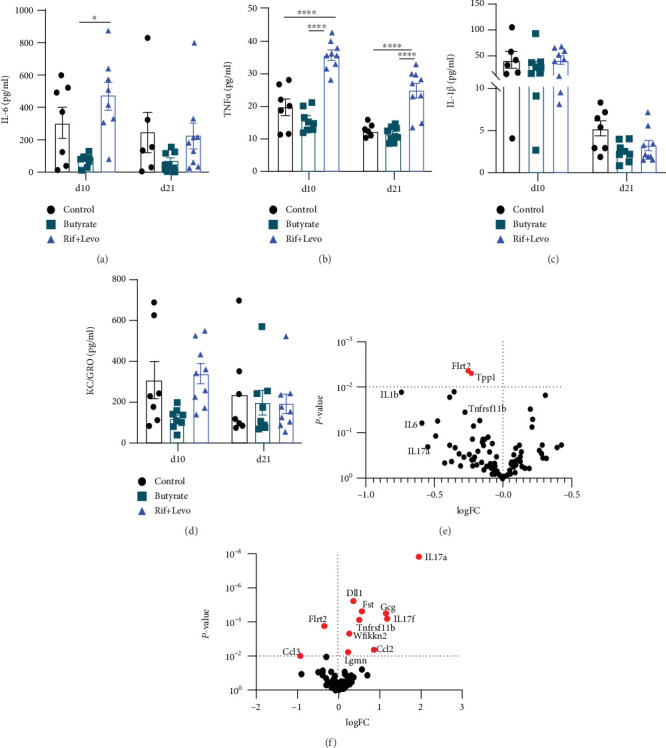
Butyrate induces a reduction of proinflammatory mediators, whereas rifampicin and levofloxacin increase proinflammatory and bone-preserving markers in serum. A variety of (pro-) inflammatory cytokines in serum of mice were assessed 10 days and 21 days following osteotomy using a multiplexed cytokine array and Olink targeted proteomics to investigate systemic biomarkers in serum of mice. Serum concentrations of (a) IL-6, (b) TNF*α*, (c) IL-1*β*, and (d) of KC/GRO (murine IL-8 homologue) in the absence and presence of butyrate and Rif + Levo, respectively, 10 days and 21 days after osteotomy. Data shown are means (*n* = 7‐9) ± SEM. Differential expression of 92 biomarkers in (e) butyrate-treated mice compared to control mice and in (f) Rif + Levo-treated mice compared to control mice. Significance threshold *p* < 0.01. Significantly changed biomarkers are labelled in red. Flrt2 = leucine-rich repeat transmembrane protein; Tpp1 = tripeptidyl-peptidase 1; Ccl3 = C-C motif chemokine 3; Lgmn = legumain; Wfikkn2 = WAP, Kazal, immunoglobulin, Kunitz, and NTR domain-containing protein 2; Tnfrsf11b = tumor necrosis factor receptor superfamily member 11B (osteoprotegerin); Ccl2 = C-C motif chemokine 2; DII1 = delta-like protein 1; Fst = follistatin; Gcg = glucagon. ⁣^∗∗∗∗^*p* < 0.0001; ⁣^∗^*p* < 0.05.

To determine the systemic effects of butyrate and antibiotic treatment, a variety of (proinflammatory) cytokines and a broad range of protein biomarkers were further assessed in the serum of mice (Figure 4). Proinflammatory IL-6 was significantly reduced in butyrate-treated mice compared to Rif + Levo-treated mice 10 days after osteotomy (*p* < 0.05), but this effect was diminished at 21 days due largely to a reduction in the levels of antibiotic-treated mice at this timepoint (Figure 4(a)). Levels of the proinflammatory TNF*α* were similar for control- and butyrate-treated mice at both timepoints; however, they were significantly increased in Rif + Levo-treated mice (Figure 4(b)). Proinflammatory IL-1*β* levels and KC/GRO, the murine IL-8 homologue, were not significantly affected at either timepoint by butyrate or antibiotic therapy (Figures 4(c) and 4(d)), although KC/GRO displayed a trend for increased levels in Rif + Levo-treated mice at 10 days (*p* = 0.0976) (Figure 4(d)). No clear differences between the groups were detected in terms of anti-inflammatory IL-10 levels at 10 days and 21 days (Supplementary Figure 3A).

Differential expression analysis of the 92 biomarkers tested with Olink® technology revealed that antibiotic treatment had a major impact on serum biomarkers with 11 proteins being significantly affected (*p* < 0.01). Butyrate induced two significant changes: Flrt2, a marker for cell-cell adhesion and migration, and Tpp1, a lysosomal serine protease, were significantly downregulated in butyrate-treated mice compared to control mice (Figure 4(e)). In addition, trends for downregulation in the proinflammatory and osteoclastogenic IL-1*β* (logFC = −0.7484; *p* = 0.0130), IL-6 (logFC = −0.6000; *p* = 0.0622), IL-17a (logFC = −0.4966; *p* = 0.01127), and Tnfrsf11b (=OPG, logFC = −0.2769; *p* = 0.0346) were observed in butyrate-supplemented mice. In contrast, IL-17a, IL-17f, and Tnfrs11b were significantly upregulated in antibiotic-treated mice compared to control mice (Figure 4(f)). Other significantly upregulated proteins were as follows: DII1, a regulator of adult stem cells; Fst (follistatin), an inhibitor of follicle-stimulating hormone (FSH); Gcg (glucagon), blood glucose regulator; Wfikkn2, a protease inhibitor; and the chemokine Ccl2. Flrt2 and the chemokine Ccl3 were significantly downregulated in antibiotic-treated mice compared to control mice. Summarizing the serum analysis, antibiotic treated, SCFA-depleted mice show higher levels of the proinflammatory and proosteoclastogenic TNF*α*, IL-17a, and IL-17f as well as higher OPG levels compared to control mice. In contrast, butyrate-treated mice show the converse trend, i.e., lower levels of proinflammatory and proosteoclastogenic IL-6, IL-1*β*, and IL-17a and lower OPG levels compared to control mice.

### 3.5. Butyrate Treatment Decreases CD14+ Monocyte/Macrophage Population but Increases CD19+ B Cell Population in the Bone Marrow of a Mice with an Osteotomy

**Figure 5:**
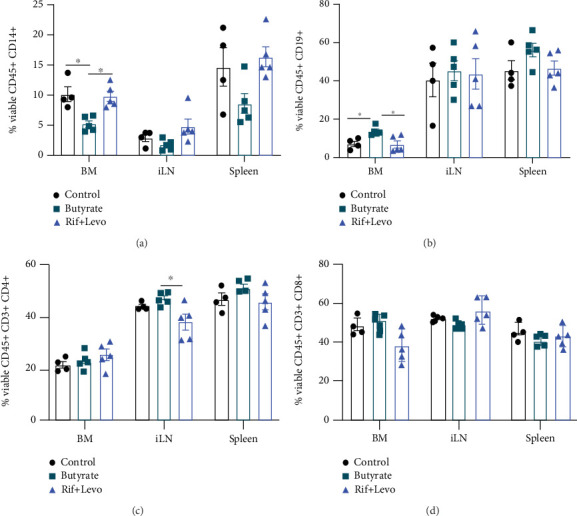
Butyrate treatment decreases the CD14+ monocyte/macrophage population but increases the CD19+ B cell population in bone marrow following osteotomy. Immunophenotyping of single-cell suspensions from bone marrow (BM), inguinal lymph node (iLN), and spleen from operated mice was performed. (a) Percent of viable CD45+ CD14+ monocytes/macrophages, (b) CD45+ CD19+ B cells, (c) CD45+ CD3+ CD4+ T cells, and (d) CD45+ CD3+ CD8+ T cells in spleen, iLN, and BM, in the absence and presence of butyrate and Rif + Levo, respectively. Data shown are means (*n* = 4‐5) ± SEM. ⁣^∗^*p* < 0.05.

We used flow cytometry to perform immunophenotyping in the spleen, the left inguinal lymph node (iLN), which drained the operated bone area, and the left tibial bone marrow (BM) of the mice. The spleen was chosen to mirror a systemic immune response, whereby BM and iLN reflect the local immune response. Cell populations of interest were gated as shown in Supplementary Figure 3B. The percent of viable CD45+ CD14+ monocyte/macrophages was reduced by ~50% in BM of butyrate-treated mice compared to both control- and antibiotic-treated mice (Figure 5(a)). Similar trends for a reduction in CD14+ populations were observed in iLN (*p* = 0.0900) and spleen (*p* = 0.0799) of butyrate-treated mice compared to Rif + Levo-treated mice. In contrast, the percentage of the viable CD45+ CD19+ B cell population in BM of butyrate-treated mice was 2-fold higher compared to control- and Rif + Levo-treated mice but remained largely unchanged in iLN and spleen (Figure 5(b)). Regarding viable CD45+ CD3+ CD4+ T cells, butyrate-treated mice showed significantly higher percentages compared to Rif + Levo-treated mice in the iLN, whereas percentages in BM spleen remained largely unchanged between groups (Figure 5(c)). Viable CD45+ CD8+ T cell percentages trended higher in butyrate-treated mice compared to Rif + Levo-treated mice (*p* = 0.0713) but did not significantly differ compared to the control group, and no large differences between groups were detected in iLN and spleen (Figure 5(d)). Taken together, butyrate mainly influences CD14+ monocytes/macrophages and CD19+ B cell population in BM of mice, therefore matching its potent effect on osteoclast precursor cells as shown *in vitro*.

### 3.6. Butyrate Does Not Significantly Impact Bone Healing Outcomes While Rifampicin and Levofloxacin May Delay Bone Healing in a Murine Osteotomy Model

**Figure 6:**
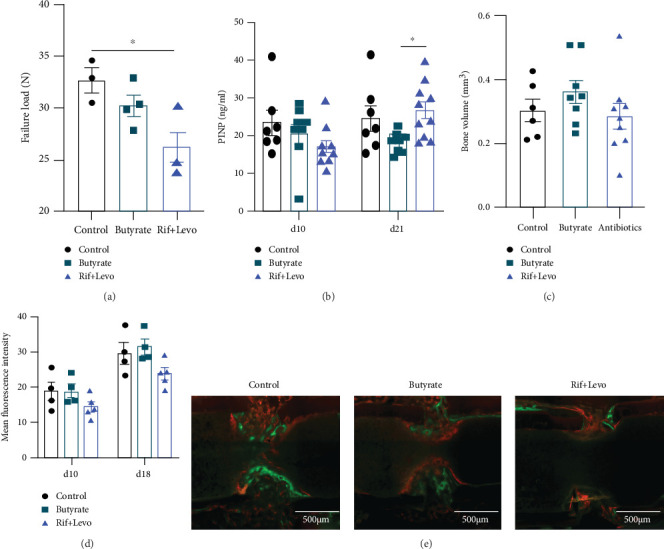
Butyrate does not significantly impact bone healing outcomes while rifampicin and levofloxacin may delay bone healing in a murine osteotomy model. Bone healing outcomes of operated mice were assessed 10 days, 18 days, and 21 days (euthanasia) following osteotomy. (a) Failure load of contralateral, unoperated femora was determined by a four-point-bending test. Data shown are means (*n* = 3‐4) ± SEM. (b) Procollagen type I C-terminal propeptide (PINP) in serum was determined 10 days and 21 days after osteotomy. Data shown are means (*n* = 7‐10) ± SEM. (c) Bone volume was measured by *μ*CT 21 days after osteotomy. Data shown are means (*n* = 6‐10) ± SEM. (d) Quantification of calcium deposition at osteotomy site 10 days after osteotomy (calcein green injection) and 18 days after osteotomy (xylenol orange injection). Data shown are mean fluorescent intensities (*n* = 4‐5) ± SEM. (e) Representative fluorescent images showing calcein green and xylenol orange labelling of exposed calcium at osteotomy site. Scale bar = 500 *μ*m, ⁣^∗^*p* < 0.05.

Based on our *in vitro* findings showing butyrate's inhibitory effect on osteoclast formation and activity and its tendency to promote osteogenic differentiation of MSCs, we assessed whether butyrate impacts bone healing in a murine osteotomy model. Bone mechanical properties of the unoperated, contralateral femur of the mice were tested to determine the failure load. Failure load of butyrate-treated mice did not significantly differ from control mice, but Rif + Levo-treated mice showed a significant (*p* < 0.05) reduction in failure load 21 days after osteotomy (Figure 6(a)). Systemic PINP, a bone turnover marker, was significantly (*p* < 0.05) higher in Rif + Levo-treated mice compared to butyrate-treated mice at 21 days, but no significant difference between the groups was detected 10 days following osteotomy (Figure 6(b)). Bone volume of the newly formed callus at the osteotomy site was quantified 21 days following osteotomy by means of *μ*CT. No significant differences between the groups could be detected in terms of newly formed bone 21 days following osteotomy (Figure 6(c)). To determine the state of mineral apposition, as an indicator for bone remodeling and healing, fluorescently labelled calcium-binding agents were administered to mice 10 days (calcein green) and 18 days (xylenol orange) after osteotomy. Rif + Levo-treated mice show a slight reduction in mean fluorescent intensity at 10 and 18 days (Figures 6(d) and 6(e)). In addition, Giemsa-Eosin staining was performed to further visualize cartilaginous and bony callus formation at the osteotomy site 21 days after surgery. Rif + Levo-treated mice show a higher abundance of cartilaginous, rather than bony, mineralized callus, compared to control- and butyrate-treated mice (Supplementary Figure 5). This is in line with the finding of reduced mean fluorescent intensity, thus mineral apposition, in the Rif + Levo-treated mice. To conclude, butyrate does not significantly affect bone properties and healing outcomes in a murine osteotomy model. However, Rif + Levo-treated mice show a trend towards delayed bone remodeling and a change in bone properties as revealed by increased PINP levels in serum, reduced mineral apposition, and failure loads 21 days following osteotomy.

## 4. Discussion

The beneficial effects of probiotics and postbiotics on a variety of inflammatory diseases such as colitis and arthritis have raised the possibility that it may also play an important role in bone fracture healing. Postbiotic SCFAs have proven capacity to resolve hyperinflammatory responses [35], which may prevent delayed fracture union, resulting from a prolonged inflammatory healing phase. Conversely, disturbances in SCFA production, for example, via antibiotic therapy, may negatively impact fracture union, although this also remains largely unstudied to date.

Our initial *in vitro* studies showed that SCFAs are potent osteoclast inhibitors, by reducing osteoclast formation and their resorption activity. Mechanistic insights into this effect were revealed by RNA sequencing, whereby several pathways crucial for bone healing were highly affected in osteoclast precursor cells after exposure to butyrate. For example, the GPR-mediated chemotaxis pathway was upregulated in the presence of butyrate. GPR41, 43, and 109a and olfactory receptor 78 are well-known receptors for SCFAs, and, of particular relevance, GPR43 (also known as FFAR2) has previously been shown to be required for the inhibitory effects of SCFAs on osteoclasts [36]. We found that stimulation of GPR43 with a selective agonist led to a significant reduction in osteoclast formation, indicating the importance of GPR43 in the prevention of osteoclast formation. Lucas et al. found that the inhibitory effect of SCFAs on osteoclasts is largely independent of GPR43 but rather occurs as a consequence of a metabolic reprograming of osteoclast precursor cells, leading to a downregulation of the essential osteoclast genes, TRAF6 and NFATc1 [37]. This discrepancy could be due to the differences in SCFA concentrations and mouse sexes used in these studies. In the context of GPR signaling, GPR183 (also known as EBI2) was shown to be required for murine osteoclast precursor migration and osteoclast differentiation [38]. Although gene expression of GPR183 was significantly downregulated upon butyrate treatment in our study, inhibition of GPR183 signaling in human osteoclast precursor cells did not affect osteoclast formation. The upregulation of Wnt5a signaling in our gene enrichment analysis indicates another link between butyrate and bone healing, since Wnt5a was shown to be upregulated in fracture repair [39]. Regarding downregulated pathways, oxidative stress-related ROS signaling was one of the most affected pathways. High oxidative stress was shown to negatively influence bone remodeling by favoring osteoclastogenesis [40]. Tang et al. further demonstrated that butyrate protected from bone loss in rats by reducing ROS levels and promoting activity of mitochondrial antioxidant enzymes [41]. Although not particularly investigated in this study, it is worth mentioning that several *in vitro* studies highlighted the inhibitory effect of butyrate on osteoclast formation being attributed to inhibitory effects on histone deacetylase (HDAC) activity [42, 43]. Based on our transcriptome data and studies from others, it becomes obvious that the SCFA-mediated inhibition of osteoclast formation is a likely consequence of effects on multiple pathways including GPR signaling, HDAC inhibition, immune-related signaling, and metabolic changes.

The effect of butyrate on osteoclast precursor cells is also apparent in our *in vivo* osteotomy model. CD14+ monocytes/macrophages, which are precursor cells for osteoclasts, were significantly decreased in the BM following butyrate administration when compared to control- and antibiotic-treated mice. It was shown previously that butyrate directly affected CD45+ CD14+ cells by reducing CD14 receptor expression through posttranscriptional mechanisms [44]. Furthermore, SCFA propionate altered bone marrow hematopoiesis by mainly affecting macrophage and dendritic cell precursors in mice [45]. The butyrate-induced reduction of monocyte/macrophages in murine bone marrow is an additional proof of the potent impact of butyrate on osteoclast formation. The effect of SCFAs, in particular butyrate, on macrophages has previously been demonstrated, whereby SCFAs increased phagocytic capacity and antimicrobial activity in those cells [46, 47]. In contrast to the reduction of the monocytes/macrophage population, butyrate increased CD19+ B cell numbers in the BM compared to control- and antibiotic-treated mice. It has been recently described that butyrate supplementation suppressed arthritis in mice in a regulatory B cell- (Breg-) dependent manner [48]. Breg cells are also crucial in the bone union process by suppressing proinflammatory cytokines [49], and loss of Breg cell function has previously been associated with delayed healing in tibial fracture patients [50]. Although we did not assess the specific contributions of Breg and Treg cells to the observed effects of butyrate in our model, this is a likely potential mediator worthy of further investigation.

Besides butyrate's impact on a variety of immune cell populations, butyrate also impacted serum inflammatory maker, notably reducing proinflammatory IL-6 levels. IL-6 is not only secreted by immune cells such as macrophages but also by osteoblasts, which then promotes osteoclast formation [51]. Interestingly, pharmacological inhibition of soluble IL-6 improved compromised fracture healing after severe trauma in mice [52]. Other cytokines, which are also known to have osteoclastogenic capacity, are TNF*α*, IL-1*β*, and IL-17 amongst others [53]. Here, Rif + Levo-treated mice show strongly increased TNF*α*, IL-17a, and IL-17f levels in the serum compared to control- and butyrate-treated mice. In fact, mice treated with Rif + Levo showed significantly reduced levels of SCFAs acetate, propionate, and butyrate in the cecal water, which was accompanied by a marked change in their gut microbiome composition [54]. The reduced levels of SCFAs might explain the high levels of circulating proinflammatory TNF*α*, IL-17a, and IL-17f levels in the serum of antibiotic-treated mice. In contrast, OPG (Tnfrsf11b) is an antiosteoclastogenic factor that preserves bone mass and is increased in antibiotic-treated mice but decreased in butyrate-treated mice compared to control mice. The correlation of TNF*α* and OPG in our study is in line with others describing TNF*α* as a promotor of OPG expression [54]. Indeed, TNF*α* was shown to upregulate OPG expression in dendritic cells, endothelial cells, smooth muscle cells, and fibroblasts [55] but also stimulated OPG secretion from human umbilical vein endothelial cells (HUVECs) [56]. Additionally, serum levels of both TNF*α* and OPG were higher in osteoarthritis patients compared to healthy patients [57]. Thus, increased expression of OPG may counteract the proosteoclastogenic effects of increased TNF*α* levels to protect from excessive bone destruction. Interestingly, OPG treatment in rats impaired callus remodeling by reducing osteoclast numbers, although it did not influence early callus expansion [58]. Also, blockade of RANK signaling with the RANKL-targeted antibody denosumab, or treatment with the bisphosphonate alendronate, was shown to delay fracture callus remodeling but improved mechanical strength and bone mineral density in mice [59, 60]. Together, this highlights that a targeted and temporary activity of osteoclasts in the fracture healing is required for successful callus remodeling. Butyrate could be a candidate compound to optimally support fracture healing by preventing overactivity of osteoclasts at the remodeling stage and to support effective resolution of the initial inflammatory phase in fracture healing.

The high levels of circulating TNF*α*, IL-6, IL-17a, and IL-17f in SCFA depleted antibiotic-treated mice are indicative of a prolonged and unresolved systemic inflammatory phase in those mice. This could explain the tendency for delayed bone healing in the antibiotic-treated mice as revealed by increased PINP levels 21 days upon osteotomy and the trend towards reduced mineral apposition at the defect site. Since PINP is produced upon cleavage of procollagen during matrix formation prior to mineralization, this could indicate that bone remodeling and mineralization is at an early stage in the antibiotic-treated mice, whereas it is further advanced in the control- and butyrate-treated mice. Furthermore, the chronic elevation of TNF*α*, IL-6, IL-17a, and IL-17f can lead to excessive and prolonged osteoclast activity, which then results in net bone loss and decreased bone stability [61]. Indeed, antibiotic-treated mice showed reduced failure load values in the contralateral, unoperated femora in this study indicating a negative systemic effect on bone turnover. The measurement of IL-17a, IL-17f, and other cytokines in this study was performed in the mid and late phase of fracture repair (10 days and 21 days upon surgery), at which the osteoclasts are key players [24]. In the early phase of fracture repair (<7 days), it has been shown that both IL-17a and IL-17f promote osteoblast maturation and accelerate osteogenesis [62, 63]. Thus, depending on the fracture healing stage, IL-17a and IL-17f might have different functions and impact on both osteoclasts and osteoblasts. Such a bimodal role of cytokines in fracture healing has also been described for IL-6 [64].

Based on our findings, an interesting aspect for further study would be to investigate if butyrate supplementation could reverse the negative effects of antibiotic treatment on bone healing. Indeed, we tested a combined regime by administrating both butyrate and antibiotics in an additional study group. However, after suffering from a greater than anticipated drop-out rate, the limited findings from this experimental group are not presented. The main reason for exclusions, which were present in all groups, was screw loosening; this may be partially attributed to the flexible fixation method and likely compounded by the use of male mice, which typically are heavier and display more pronounced barbering behavior than female mice. Flexible fixation methods are known to induce higher inflammatory callus formation and so were selected for this study to reveal the potential for SCFAs to modulate healing in a positive direction. In order to test this combination of butyrate and antibiotics, refinement of the model may be required, through the use of a more rigid fixation approach, for example. The impact of butyrate in combination with antibiotics may also be tested in a bone infection model where significant inflammatory osteolysis is expected.

## 5. Conclusion

In conclusion, butyrate significantly reduced osteoclast formation and resorption activity in a dose-dependent manner and displayed a trend for increased calcium deposits in MSC cultures. *In vivo*, butyrate reduced monocyte/macrophages in the bone marrow and systemic IL-6 levels in a murine osteotomy model compared to control- and antibiotic-treated mice. In contrast, antibiotic-treated mice showed reduced SCFA levels in the cecum and higher circulating proinflammatory TNF*α*, IL-17a, and IL-17f levels. Antibiotic-treated mice also displayed a trend towards delayed bone healing compared to control- and butyrate-treated mice.

This study highlights the potential of the gut microbiota and its associated SCFAs as potential contributors to successful bone healing.

## Data Availability

Fastq data files of RNA sequencing and 16s rRNA sequencing will be deposited on http://ncbi.nlm.nih.gov/ upon manuscript publication. Other data is available upon request to authors.

## Supplementary Materials

Supplementary Materials Supplementary Figure 1: (A, B) impact of acetate, propionate, butyrate, valerate (A), and antibiotics (B) on osteoclast formation. Osteoclast precursor cells were cultured with 20 ng/mL M-CSF and 10 ng/mL RANKL and with 0.1 mM, 0.25 mM, 0.5 mM, or 1 mM of the different SCFA classes, or 0.5 *μ*g/mL, 5 *μ*g/mL, or 50 *μ*g/mL rifampicin and/or levofloxacin, respectively. Osteoclast formation was quantified by means of TRAcP staining. Shown are means (*n* = 3 independent donors, triplicates per donor) ± SEM. (C) MSCs were cultured in chondrogenic media, and sulphated glycosaminoglycan (sGAG) content in cell pellets was assessed and normalized to DNA content. Data shown are means (*n* = 5 independent donors) with ±SEM. Representative images of Safranin O and Fast Green staining in the absence (left image) and presence of 0.5 mM butyrate (right image). (D, E) Effects of butyrate on cell viability of osteoclast precursors (D) and MSCs (E) were assessed using CellTiter-Blue reagent. Data shown are means (*n* = 3 independent donors, triplicates per donor) ± SEM. Percent cell viability was normalized to untreated osteoclast precursor cells and MSCs, respectively. ⁣^∗∗∗∗^*p* < 0.0001, ⁣^∗∗∗^*p* < 0.001, ⁣^∗∗^*p* < 0.01, and ⁣^∗^*p* < 0.05. Supplementary Figure 2: (A, B) impact of GPR43 agonist and GPR183 inverse agonist (inhibitor) on osteoclast formation. Human osteoclast precursor cells were cultured with 20 ng/mL M-CSF and 10 ng/mL RANKL and with 75 nM, 750 nM, or 7500 nM GPR43 agonist (A) or 5 nM, 50 nM, or 500 nM inverse GPR183 agonist (B). Osteoclast formation was quantified by means of TRAcP staining. Shown are means (*n* = 3 independent donors, triplicates per donor) ± SEM. ⁣^∗∗^*p* < 0.01; UT = untreated; RKL = RANKL. (C) Representative images of TRAcP staining of untreated (UT), RANKL-stimulated cells (RKL), and upon treatment with either 500 nM GPR183 inhibitor or 7500 nM GPR43 agonist. Scale bar in all images = 100 *μ*m. Supplementary Figure 3: (A) Principle Coordinate Analysis (PCoA) was performed on filtered ribosomal sequence variants (RSVs) using Bray-Curtis distances. (B) Percent abundances of 50 most abundant RSVs grouped by taxonomic family. (C) Heat map of the 50 most abundant genera as assigned by DADA2 analysis pipeline. Supplementary Figure 4: (A) serum concentrations of IL-10 in the absence and presence of butyrate and Rif + Levo, respectively, 10 days and 21 days upon osteotomy. (B) Gating strategy of flow cytometric immunophenotyping. Viable (eFluor 780 negative cells) were selected, and CD45+ cells were further gated from this population. CD19+, CD14+, CD3+ CD4+, and CD3+ CD8+ cells were selected as presented. Supplementary Figure 5: representative images of osteotomy sites 21 days after surgery stained with Giemsa-Eosin. Images were chosen based on bone volume median of each treatment group measured by *μ*CT 21 days upon surgery. Scale bar = 500 *μ*m.
