# Supplementary figures and images for: Butyrate Inhibits Osteoclast Activity In Vitro and Regulates Systemic Inflammation and Bone Healing in a Murine Osteotomy Model Compared to Antibiotic-Treated Mice
Source: Mediators Inflamm. 2021 Dec 10;2021:8817421. doi: 10.1155/2021/8817421 (PMC8683197; doi:10.1155/2021/8817421)

Supplementary Figure 1

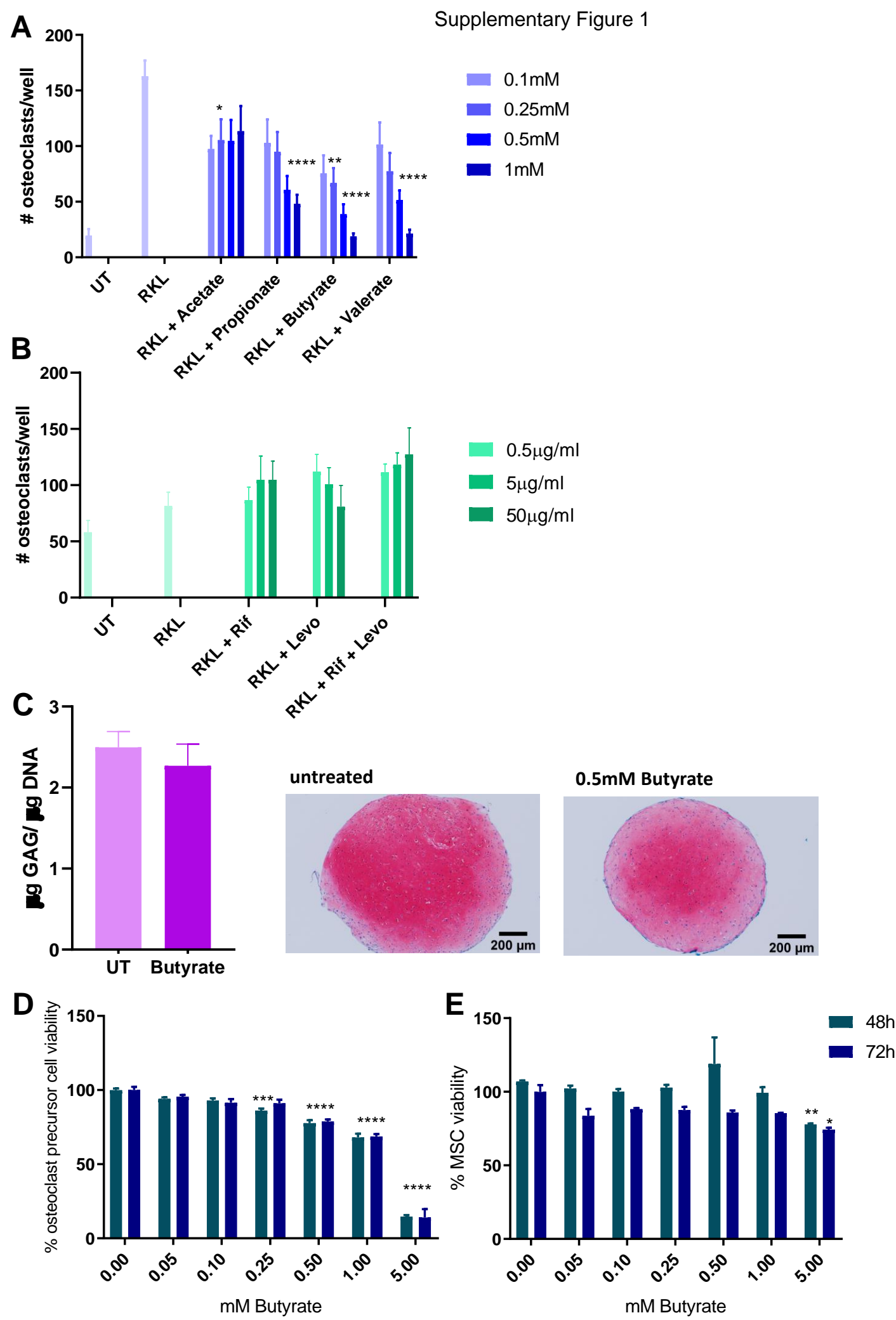

**A**

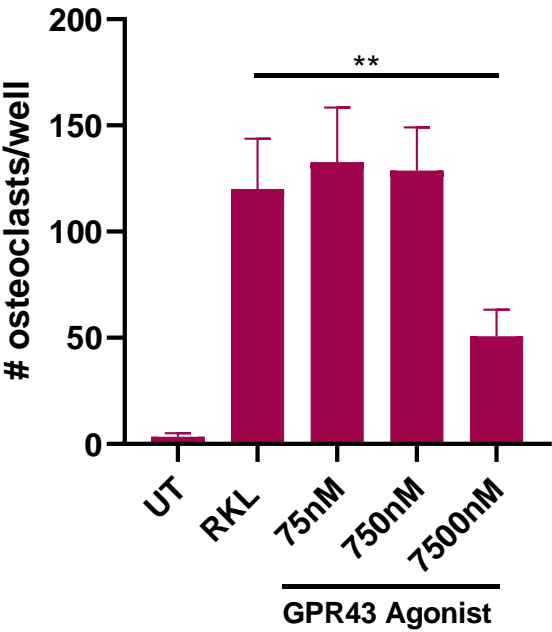

**B**

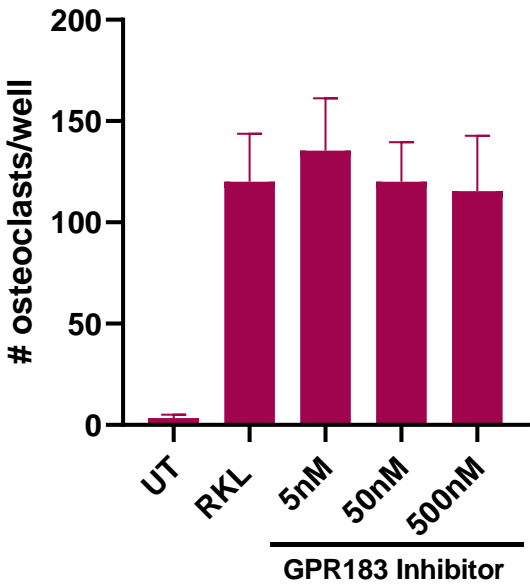

**C**

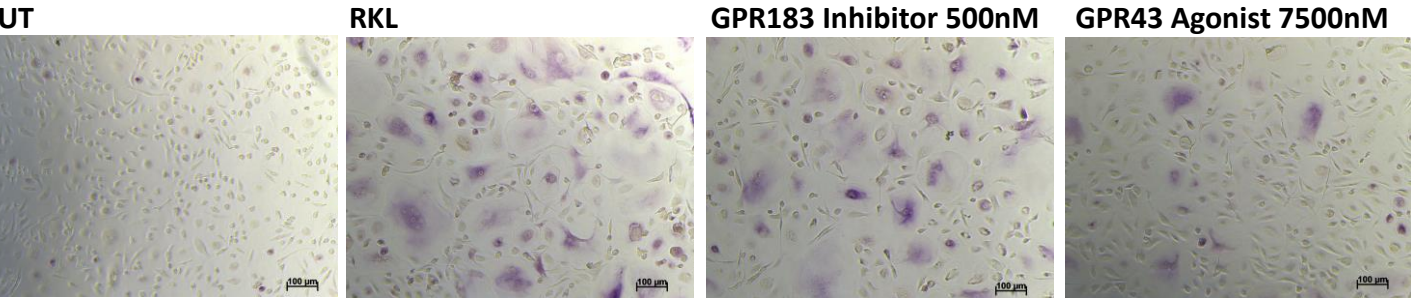

Supplementary Figure 3

A

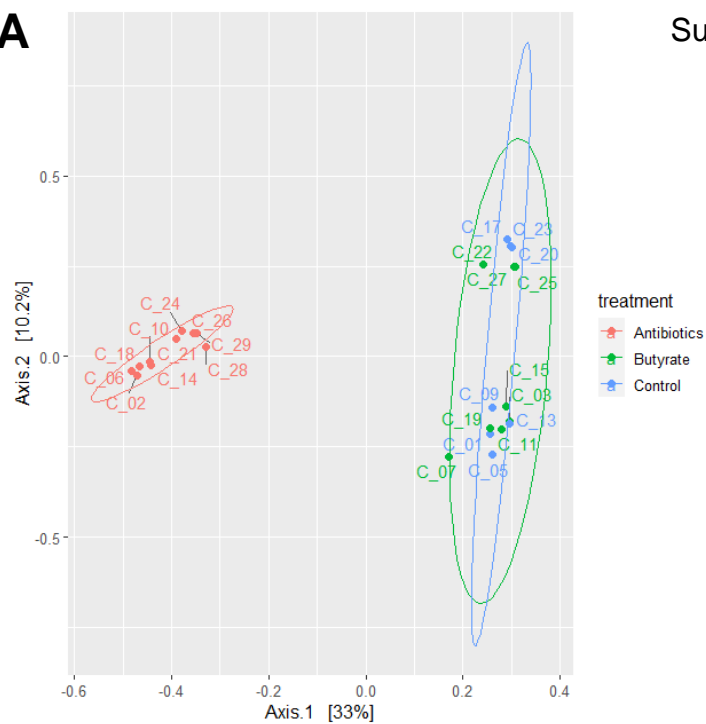

B

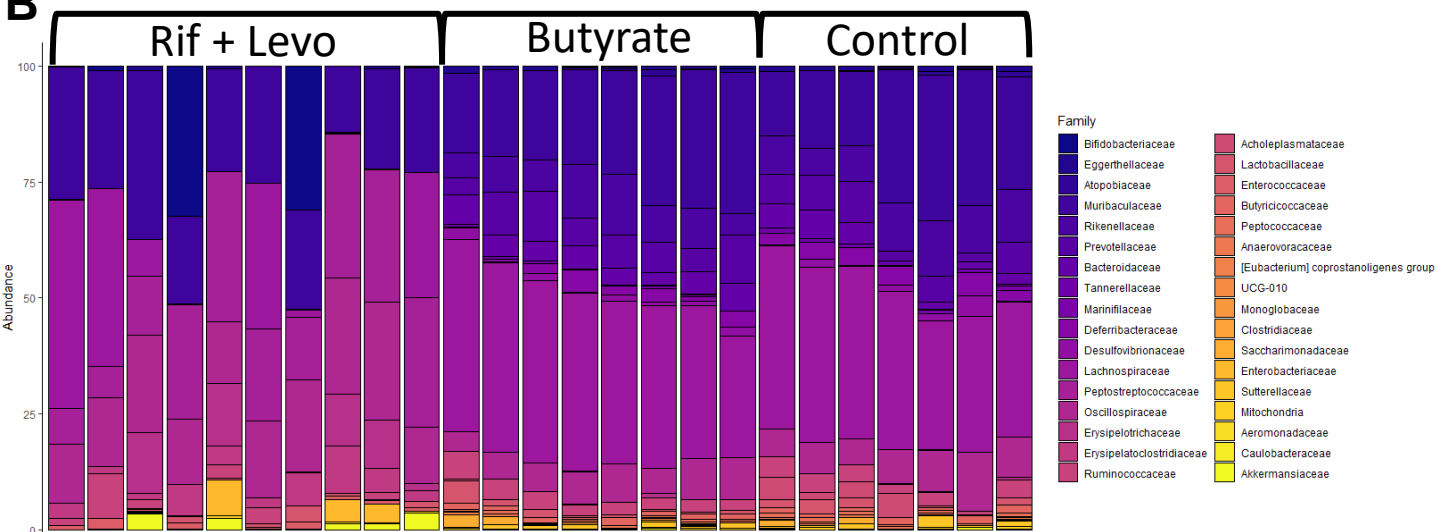

C

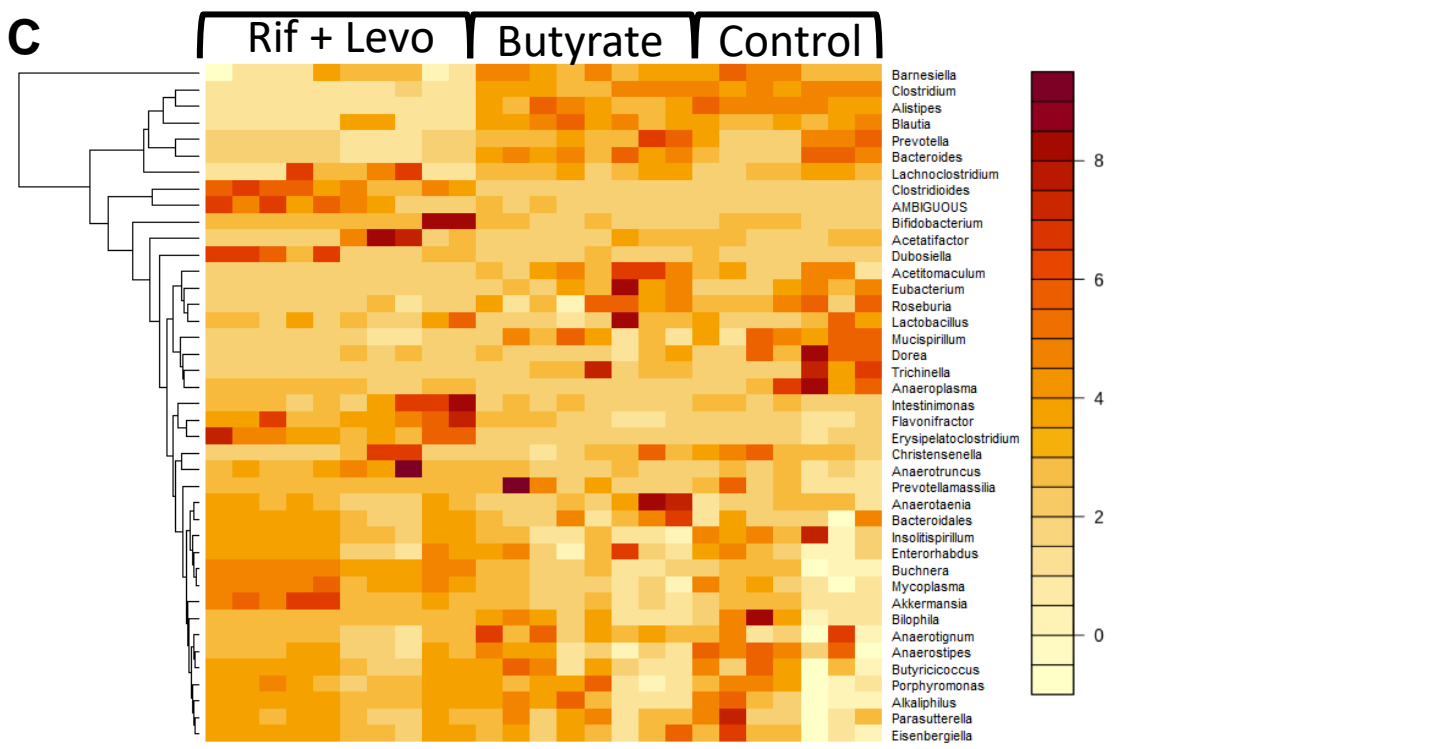

**A**

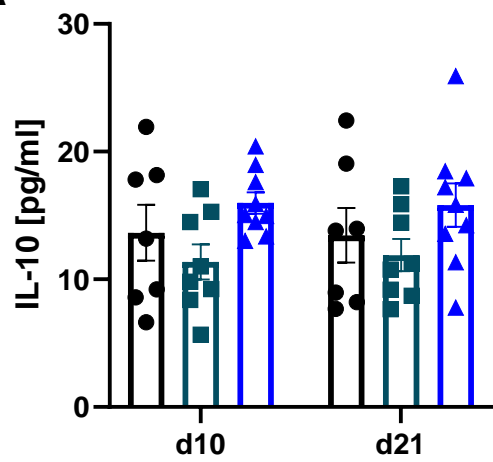

**B**

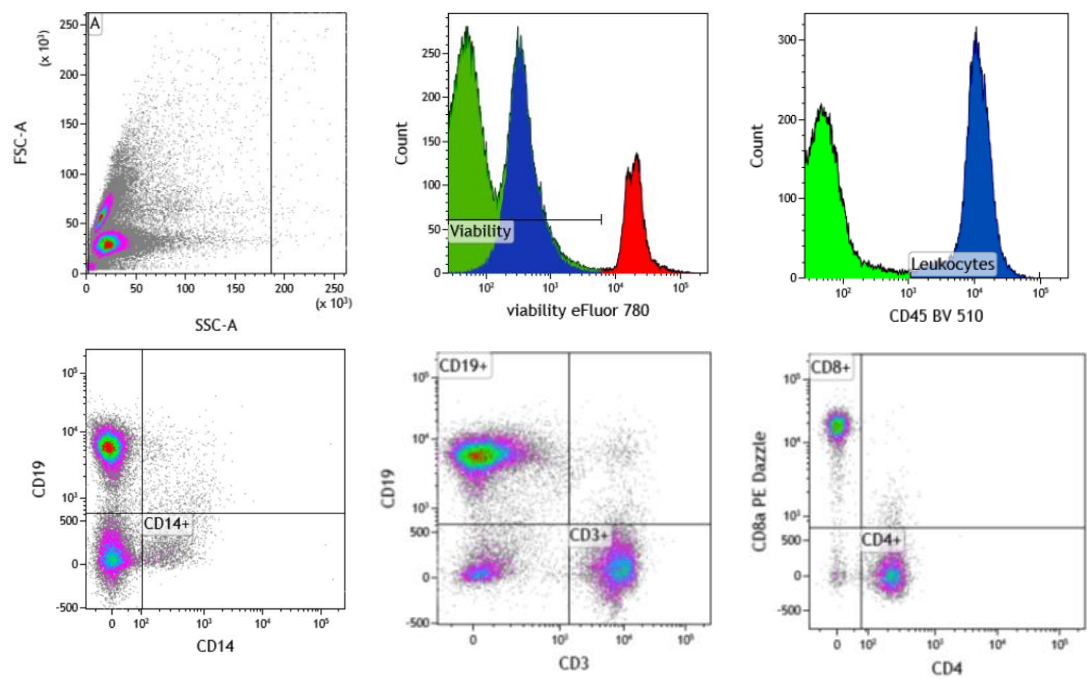

**Control**

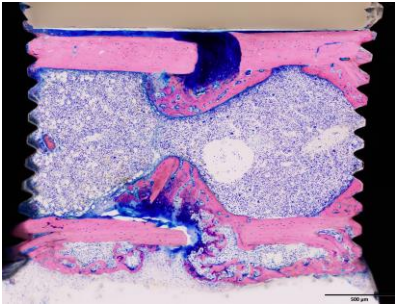

**Butyrate**

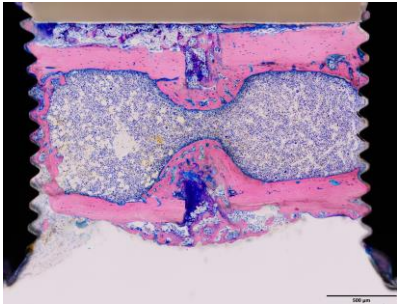

**Rif + Levo**

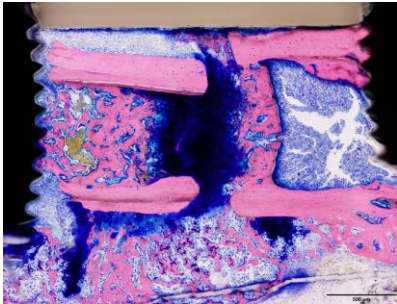

Supplement: Supplementary Materials — Supplementary Figure 1: (A, B) impact of acetate, propionate, butyrate, valerate (A), and antibiotics (B) on osteoclast formation. Osteoclast precursor cells were cultured with 20 ng/mL M-CSF and 10 ng/mL RANKL and with 0.1 mM, 0.25 mM, 0.5 mM, or 1 mM of the different SCFA classes, or 0.5 μg/mL, 5 μg/mL, or 50 μg/mL rifampicin and/or levofloxacin, respectively. Osteoclast formation was quantified by means of TRAcP staining. Shown are means (n = 3 independent donors, triplicates per donor) ± SEM. (C) MSCs were cultured in chondrogenic media, and sulphated glycosaminoglycan (sGAG) content in cell pellets was assessed and normalized to DNA content. Data shown are means (n = 5 independent donors) with ±SEM. Representative images of Safranin O and Fast Green staining in the absence (left image) and presence of 0.5 mM butyrate (right image). (D, E) Effects of butyrate on cell viability of osteoclast precursors (D) and MSCs (E) were assessed using CellTiter-Blue reagent. Data shown are means (n = 3 independent donors, triplicates per donor) ± SEM. Percent cell viability was normalized to untreated osteoclast precursor cells and MSCs, respectively. ⁣∗∗∗∗p < 0.0001, ⁣∗∗∗p < 0.001, ⁣∗∗p < 0.01, and ⁣∗p < 0.05. Supplementary Figure 2: (A, B) impact of GPR43 agonist and GPR183 inverse agonist (inhibitor) on osteoclast formation. Human osteoclast precursor cells were cultured with 20 ng/mL M-CSF and 10 ng/mL RANKL and with 75 nM, 750 nM, or 7500 nM GPR43 agonist (A) or 5 nM, 50 nM, or 500 nM inverse GPR183 agonist (B). Osteoclast formation was quantified by means of TRAcP staining. Shown are means (n = 3 independent donors, triplicates per donor) ± SEM. ⁣∗∗p < 0.01; UT = untreated; RKL = RANKL. (C) Representative images of TRAcP staining of untreated (UT), RANKL-stimulated cells (RKL), and upon treatment with either 500 nM GPR183 inhibitor or 7500 nM GPR43 agonist. Scale bar in all images = 100 μm. Supplementary Figure 3: (A) Principle Coordinate Analysis (PCoA) was performed o [file 8817421.f1.pdf]
